# B Vitamins and Their Role in Immune Regulation and Cancer

**DOI:** 10.3390/nu12113380

**Published:** 2020-11-04

**Authors:** Christine Tara Peterson, Dmitry A. Rodionov, Andrei L. Osterman, Scott N. Peterson

**Affiliations:** 1 Center of Excellence for Research and Training in Integrative Health, Department of Family Medicine and Public Health, UC San Diego, School of Medicine, La Jolla, CA 92093, USA; chpeterson@ucsd.edu; 2 Bioinformatics and Structural Biology Program, Sanford Burnham Prebys Medical Discovery Institute, La Jolla, CA 92037, USA; rodionov@SBPdiscovery.org; 3 A.A. Kharkevich Institute for Information Transmission Problems, Russian Academy of Sciences, 119991 Moscow, Russia; 4 Immunity and Pathogenesis Program, Infectious and Inflammatory Diseases Center, Sanford Burnham Prebys Medical Discovery Institute, La Jolla, CA 92037, USA; osterman@SBPdiscovery.org; 5 Tumor Microenvironment and Cancer Immunology Program, Sanford Burnham Prebys Medical Discovery Institute, La Jolla, CA 92037, USA

**Keywords:** thiamine, riboflavin, niacin, pantothenic acid, pyridoxine, biotin, folate, cobalamin, gut microbiota, inflammation, oxidative stress, cancer

## Abstract

B group vitamins represent essential micronutrients for myriad metabolic and regulatory processes required for human health, serving as cofactors used by hundreds of enzymes that carry out essential functions such as energy metabolism, DNA and protein synthesis and other critical functions. B vitamins and their corresponding vitamers are universally essential for all cellular life forms, from bacteria to humans. Humans are unable to synthesize most B vitamins and are therefore dependent on their diet for these essential micronutrients. More recently, another source of B vitamins has been identified which is derived from portions of the 10^13^ bacterial cells inhabiting the gastrointestinal tract. Here we review the expanding literature examining the relationship between B vitamins and the immune system and diverse cancers. Evidence of B vitamin’s role in immune cell regulation has accumulated in recent years and may help to clarify the disparate findings of numerous studies attempting to link B vitamins to cancer development. Much work remains to be carried out to fully clarify these relationships as the complexity of B vitamins’ essential functions complicates an unequivocal assessment of their beneficial or detrimental effects in inflammation and cancers.

## 1. Introduction

B vitamins (B1, thiamine, B2, riboflavin, B3, niacin, B5, pantothenic acid B6, pyridoxine, B7, biotin B9, folate and B12, cobalamin) are essential micronutrients for all cellular life. The essentiality of B vitamins stems from the fact that B vitamins are key intermediates of pathways that generate essential cofactors such as: B1, Thiamine PyroPhosphate (TPP), B2, Flavin MonoNucleotide/Flavin Adenine Dinucleotide (FMN/FAD), B3, Nicotinamide Adenine Dinucleotide (NAD), B5, Coenzyme A (CoA), B6, PyroxidaL Phosphate (PLP), B7, Biotin-Adenine MonoPhosphate (biotin-AMP) and B9, TetraHydroFolate (THF) and B12, cobalamin that are required for hundreds of enzymes carrying out critical cellular functions. The pleiotropic nature of B vitamins emphasizes their importance to cell function and homeostasis but also complicates studies aimed at determining the contexts where B vitamins promote beneficial or detrimental effects on human health, when B vitamins are in insufficient or excessive supply. The World Health Organization (WHO) provides guidelines for the minimal daily consumption of B vitamins in the adult diet (B1: 1.1–1.2 mg, B2: 1.0–1.3 mg, B3: 11–12 mg, B5: 5 mg, B6: 1.3–1.7 mg, B7: 30 μg, B9: 400 μg and B12: 2.4 μg). The variation in B vitamin demands represents a complex relationship between the absorption, stability and number of enzymes requiring these cofactors.

Dietary B vitamins are supplied by plants and animal products in ample quantities, although a variety of conditions can increase the demand and cause deficiencies in B vitamins. Malnutrition, which remains a prevalent problem in developed and developing countries world-wide [1], exercise, stress, drug abuse, alcohol consumption and pregnancy also increase cellular demands for B vitamins [2]. Notably, vitamin B12 is not produced by plants, placing vegans and vegetarians solely reliant on microbial sources, thereby placing them at risk for B12 deficiency in the absence of supplementation. In the following sections, we describe a newly discovered source of B vitamins, as well as experimental and clinical findings that relate each B vitamin to its role in immune regulation and the related area of cancer.

## 2. Gut Microbes Generate B Vitamins.

Beyond acquisition of B vitamins from the diet, the gut microbiota is now recognized as a potential source of B vitamins. One useful distinction between dietary and microbial B vitamins is that dietary B vitamins are absorbed most prominently in the small intestine, whereas the majority of bacterial-generated B vitamins are produced and absorbed in the large intestine. It remains unclear whether these distinct sites of B vitamin absorption have any differential effects on the host. It is conceivable that gut immune cell populations that are differentially represented in the small and large intestine may be differentially activated/repressed in a site-specific manner, although direct evidence of this possibility remains unverified. Numerous species resident in gut microbiota synthesize B vitamins. Perhaps surprising, not all gut microbes are prototrophic for B vitamin biosynthesis, despite their essentiality for cellular life. Indeed, B vitamin auxotrophs for vitamins B1, B2, B3, B5 and B9 represent ~20–25% of bacterial communities, whereas B7 and B12 auxotrophy is more prevalent comprising 30 and >50% of the microbial community, respectively [3]. Given the universal essentiality of B vitamin-derived cofactors, the presence of auxotrophs led us to test the hypothesis that B vitamin prototrophs must share B vitamins with auxotrophs and presumably also with their host. Gnotobiotic mice colonized with human fecal material were provided diets lacking B vitamins or containing them in normal quantities or in 30-fold excess. Surprisingly, after 4 weeks on any of these diets, the percentage of auxotrophs for each B vitamin remained unchanged, suggesting that B vitamin sharing is able to sustain the relative fitness of B vitamin auxotrophs and that the quantities shared were not limiting [4]. It remains unclear whether the microbial provision of B vitamins is through active secretion or more trivially due to cell lysis or whether the supply of B vitamins is sufficient to sustain the health and fitness of their host in the absence of adequate dietary supplementation. The occurrence of B vitamin deficiencies in human populations, would suggest that microbial B vitamin provisions are insufficient in the long-term to sustain health.

**Figure 1:**
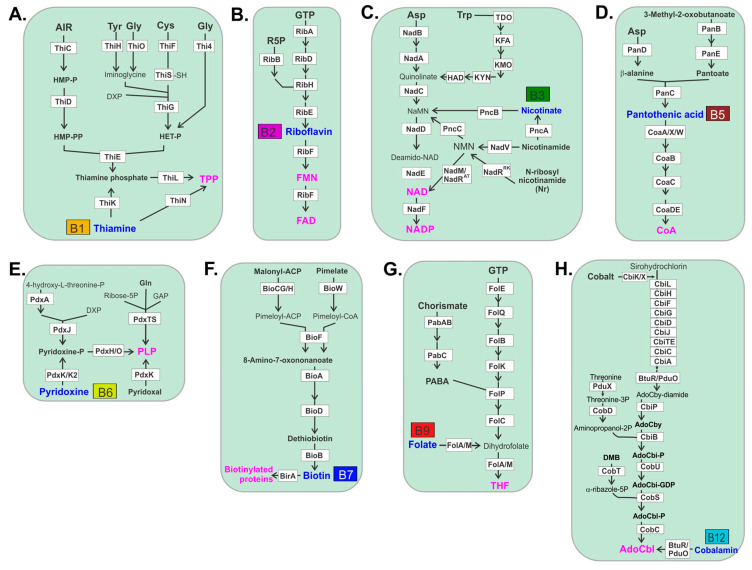
Biochemical pathways for vitamin/cofactor biosynthesis in human gut microbiome. (**A**) thiamine pyrophosphate (TPP); (**B**) Flavin MonoNucleotide/Flavin Adenine Dinucleotide FMN/FAD; (**C**) Nicotinamide Adenine Dinucleotide (Phosphate) NAD/NADP; (**D**) coenzyme A (CoA); (**E**) pyridoxal-phosphate (PLP); (**F**) biotin; (**G**) tetrahydrofolate (THF); (**H**) adenosylcobalamine (AdoCbl). Enzymes are shown in white boxes. B-vitamins/cofactors are in blue/magenta text.

Bacterial biosynthesis of thiamine is complex and may be initiated using a number of amino acid substrates such as tyrosine, glycine or cysteine (Figure 1). Biosynthesis of riboflavin uses ribose-5-phosphate and GTP as substrates. Riboflavin is then converted in a single-step to the active cofactors, FMN and FAD generated by riboflavin kinase and FAD synthase, respectively, that act as electron carriers for a number of critical redox reactions. Bacterial synthesis of niacin and its resulting cofactors utilizes aspartic acid or tryptophan to form quinolate. Nicotinate, nicotinamide or *N*-ribosyl nicotinamide that is converted to nicotinic acid mononucleotide is subsequently used to form NAD in a two-step reaction. Bacterial biosynthesis of pantothenic acid is initiated with aspartic acid that is converted to β-alanine, which is then linked to pantoate and subsequently converted to pantothenate. Pantothenate is then converted in a multistep reaction to CoA. Bacterial biosynthesis of pyridoxine is the simplest vitamin production pathway that uses either 4-hydroxyl-l-threonine-phosphate or through an alternative route, combines ribose 5-phosphate, glutamine and glyceraldehyde phosphate as substrates. Bacteria synthesize biotin through one of two primary pathways, in one malonyl-acyl carrier protein (ACP) is converted to malonyl-ACP methyl ester and subsequently converted to pimeloyl-ACP methyl ester which may also be used as substrate directly to produce pimeloyl-ACP. A third pathway uses pimelate as substrate that is converted to pimeloyl-CoA. These pathways then converge in a four-step reaction to form biotin. Folate is produced by bacteria from chorismate and GTP as precursors. Chorismate is converted to 4-aminobenzoic acid (PABA) in a two-step reaction where it combines the GTP arm of the pathway to form THF. Cobalamin biosynthesis is by far the most gene-intensive pathway used by bacteria for any B vitamin, requiring 23 separate genes. This may be the reason why B12 auxotrophs are significantly more prevalent compared to any other B vitamin produced by bacteria.

## 3. Thiamine B1

**Figure 2:**
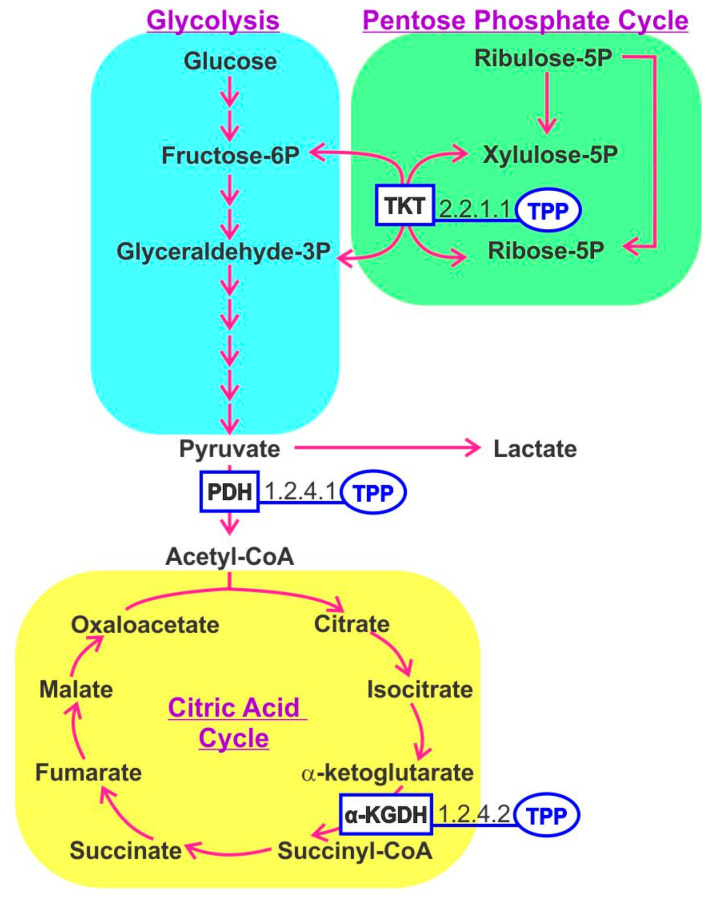
The central carbohydrate metabolism pathways involving thiamine pyrophosphate (TPP)-dependent enzymes. TPP serves as a cofactor for steps regulating the pentose phosphate cycle, fate of pyruvate and the tricarboxylic acid cycle. TKT; transketolase, PDH; pyruvate dehydrogenase, KGDH; α-ketoglutarate dehydrogenase.

In the human host, thiamine-derived molecules serve as an important cofactor for numerous enzymes, notably those involved in energy production via the tricarboxylic acid (TCA) cycle [5]. The active form of thiamine is thiamine pyrophosphate (TPP). Within mitochondria, pyruvate dehydrogenase and α-ketoglutarate dehydrogenase utilize TPP as a cofactor, whereas TPP is also important in converting amino acids and fatty acids to Acetyl CoA. TPP is a cofactor for transketolase that generates ribose-5-phosphate for RNA and DNA synthesis. A recent concept considers energy metabolism and the balance between glycolysis and TCA cycle activities as a determinant controlling immune cell function, referred to as immunometabolism [6]. Thiamine-derived cofactors play an important role in enzymes involved in these pathways (Figure 2). T-regulatory cells (Tregs), resting macrophage and naïve T cells generate energy mostly through the TCA cycle, whereas activated macrophage and Th1, Th2 and Th17 cells shift the balance toward aerobic glycolysis to complement energy derived from the TCA cycle [7]. This phenomenon has also been documented for B cells [8]. The relevance of B1 in immunometabolism was illustrated in mice fed a B1-deficient diet that curtailed the maintenance of naïve B cells but had little effect on differentiated IgA producing plasma cells [9]. This mirrors the behavior of malignant cell growth that reprograms its metabolism to favor glycolysis in the so-called Warburg effect. 

In mice, varying doses of thiamine (50, 100 and 125 mg/kg) were injected intraperitoneally in the context of xylene-induced edema. All doses of thiamine were analgesic with higher doses displaying a more prolonged effect. Similarly, edema was curtailed by all doses of thiamine indicating an anti-inflammatory effect in that setting [10]. Thiamine deficiency is a well-established feature of neurodegenerative diseases such as Alzheimer’s Disease (AD). Thiamine deficiency in this model shows congruent patterns of neuronal death occurring in brain regions displaying reduced thiamine-dependent enzyme activities [11]. Thiamine deficiency induced ferritin expression in activated microglia in vulnerable regions of the brain. Microglial cells are loaded with redox active iron that may directly contribute to oxidative stress and neuronal death in AD [12]. Thiamine–immune cell interactions are mediated by hemin-dependent oxygenases that impact the release of IntraCellular Adhesion Molecules (ICAMs) which serve to localize cells expressing various integrins [13]. Thiamine has antioxidative effects on neutrophils that protect sulfhydryl groups on the cell surface [14]. In macrophages, thiamine suppresses oxidative stress-induced activation of NF-κB and pro-inflammatory cytokine release [15].

Multiple reports have examined the effect of thiamine as part of a regimen to treat acute sepsis in conjunction with hydrocortisone and ascorbic acid [16]. Death due to sepsis is regarded as a consequence of the host response rather than the infection per se. Thiamine deficiency in septic patients is prevalent (20–70%) and thought to contribute to a variety of phenotypes associated with septic shock including decreased ATP production and increased Reactive Oxygen Species (ROS) [17]. A study of 88 patients with sepsis showed that the thiamine treatment group had significantly lower levels of lactate indicating a restoration of mitochondrial function and ATP production and corresponding reductions in mortality rates [17]. Additional studies are required to clarify the effects of thiamine on immune cell functions and inflammation. 

The speculation of a role for thiamine in cancer stems from its role in mediating enzyme activities that are relevant to tumor cells’ increased proliferation and accompanying energy demands. Further investigation regarding thiamine supplementation to support nutrition is warranted, given thiamine’s unknown oncogenic potential. This consideration highlights an important unanswered question for all B vitamins that should strive to distinguish protective effects in healthy individuals distinctly from their role in cancer patients, where B vitamins may be therapeutic or contribute to oncogenesis. 

An in vitro study using the breast cancer cell line MCF7, compared to the non-tumorigenic line MCF10A, treated with various doses of thiamine showed that high doses (1 mg and 2 mg/mL for 24 h) significantly reduced cell proliferation in MCF7 cells only. This reduction was associated with reduced glycolysis and activation of the pyruvate dehydrogenase (PDH) complex [18]. The hypoxia-inducible factor Hif-1α increases thiamine uptake in tumor cells under hypoxic conditions [19]. In a study involving seven tumor cell lines under hypoxic conditions, thiamine pyrophosphate kinase-1 (TPK1) that converts thiamine to TPP is upregulated. Despite the upregulation of TPK1, intracellular TPP was decreased. SiRNA knockdown of TPK1 reduced tumor cell proliferation, suggesting that increased TPP supports increased tumor growth [20]. A study motivated by the observation of poor thiamine bioavailability in tumors developed thiamine mimetics to improve delivery to cells. Administration of these mimetics increased intracellular TPP and reduced PDH phosphorylation by PDK while reducing tumor cell proliferation. PDK is over-expressed in tumor cells and phosphorylation of PDH drives aerobic glycolysis to complement TCA cycle energy generation. One of the mimetics tested (benfotiamine but not sulbutiamine) reduced growth of subcutaneous xenograft tumors in mice [21].

Using Erlich’s ascites tumor model, administration of 25-fold excess compared to FDA guidelines for thiamine increased tumor growth; however, at 2500 times the recommended daily allowance, modest tumor growth inhibition was observed. This attenuation of tumor growth was enhanced when high-dose thiamine was administered 7 days prior to tumor inoculation [22]. Taken together, these results suggest that thiamine may promote or inhibit tumorigenesis depending on the cancer type analyzed or the effective dose or amount of intracellular TPP.

Thiamine supplementation has been investigated with respect to cancer risk and its effect on tumorgenicity. The results of studies are mixed, some studies showing protective effects and others no significant differences. A study of an Italian ORDET cohort including 391 breast cancer cases and B vitamin intake indicated that, overall, thiamine was protective and particularly protective in estrogen- and progesterone receptor-negative HER2-positive cases [23]. By contrast, a similar study examining a broad set of cancers in Canadian women failed to show a significant correlation between thiamine intake and cancer risk [24]. A study conducted in rats examined the effect of a reduced thiamine diet and found that thiamine deficiency was associated with increased frequency of aberrant crypt foci in the colon [25].

Both in vitro and in vivo studies generate disparate findings with respect to thiamine’s role in cancer and may be nuanced and dependent on cancer subtypes. Taken together, current knowledge suggests that additional work should be undertaken to resolve apparent disagreements between studies and analyze thiamine’s impact on cancer at a higher resolution involving cancer subtypes. 

## 4. Riboflavin B2

In humans, several FAD-dependent enzymes, such as glutathione reductase, that facilitate the redox cycle of glutathione point to riboflavin’s role as a regulator of oxidative stress. Indeed, riboflavin deficiency, clinically referred to as ariboflavinosis, leads to elevated oxidative stress [26]. Riboflavin protects against oxidant-mediated inflammatory injury in lungs [27]. Detailed biochemical analyses have shown that riboflavin or FAD plays a central role in regulating the activity of phagocytic NADPH oxidase that generates superoxide anions in response to infection [28]. Riboflavin activates phagocytosis and proliferation of macrophages and neutrophils [29]. Interestingly, riboflavin inhibited neutrophil migration and infiltration and accumulation of activated granulocytes at peripheral sites that may decrease inflammatory responses [30]. Riboflavin inhibits the lipopolysaccharide (LPS)-induced ROS that leads to activation of NF-kB, thereby downregulating TNF-α and NO in a variety of experimental models [31]. A study involving undifferentiated pro-monocytic lymphoma cells demonstrated that riboflavin did not impact cell proliferation or apoptosis. Riboflavin did significantly decrease cell migration in a trans-well assay and also downregulated the expression of the checkpoint protein PD-L1 that downregulates cytotoxic CD8^+^ T cell effector functions [32].

Among a variety of immune modulatory effects hypothesized for riboflavin, the most intensively studied is its role in activation of Mucosal-Associated Invariant T (MAIT) cells. MAIT cells are abundant innate-like lymphocytes that predominantly reside in barrier tissues, where microbes coexist. Indeed, it is likely that MAIT cells’ primary function may be to protect the host from bacterial infection. Germ-free mice have very low MAIT cell abundance compared to conventional mice [33]. MAIT cell ligands include riboflavin-derived molecules that are presented by MR1, a non-polymorphic major histocompatibility complex class I-like molecule [34]. A study examining a panel of *S. pneumoniae* serotype 19A isolates assessed the activation of MAIT cells and effector functions [35]. Human monocyte-derived dendritic cells (DCs) infected with *S. pneumoniae* isolates displayed high variability in MAIT cell activation. Attempts to analyze metabolites tracked with MAIT cell activation efficiency were carried out. Among the known MAIT cell ligands are precursors of riboflavin 6,7-dimethyl-8-ribityllumazine (DMRL) and the downstream gene product, 5-amino-6-ribityluracil (5-A-RU). 5-A-RU is the most potent MAIT cell ligand [34,36]. 5-A-RU can be linked to either glyoxal or methylglyoxal to form additional ligands. ribD encodes a pyrimidine deaminase/reductase which generates 5-A-RU. Indeed, *S. pneumoniae* ribD knockout strains were unable to activate MAIT cells.

A number of studies indicate that riboflavin reduces mortality in LPS-induced septic shock by reducing a number of inflammatory cytokines, such as IL-1, IL-1β, IL-6, IFN-γ and NO, in mice [37]. In addition, riboflavin is involved with reduced oxidative stress via increased expression of inducible nitric oxide synthase (iNOS) and catalase [38]. The optimization of ROS in combatting bacterial infections by *Listeria monocytogenes* and *S. aureus* is dependent on riboflavin [39].

Riboflavin reversed liver cancer progression in animal models using carcinogenic inducers. The mechanism proposed for this effect was due to increased expression of apoptotic genes and decreases in antiapoptotic factors [40]. Studies in riboflavin-deficient rats treated with *N*-nitrosomethylbenzene to induce esophageal tumors showed increased chronic inflammation-associated genomic instability, which was elevated compared to control animals provided a normal riboflavin diet [41]. Riboflavin inhibited melanoma metastasis to the lung in animal models [42].

Riboflavin deficiency has been defined as a risk factor for cancer in general, although some data are conflicting in this regard [43]. A meta-analysis of 27 studies involving nearly 50,000 cases and over 1.2 million individuals analyzing riboflavin and other B vitamins concluded that B2 might decrease the risk of breast cancer, although significance was borderline [44]. Another meta-analysis examining components of one-carbon metabolism and the risk of renal carcinoma found no significant correlations with any B vitamins and cancer risk [45]. Riboflavin concentrations in the blood of patients with esophageal squamous cell carcinoma is reduced compared to healthy subjects [46]. Similar reports suggest that riboflavin may decrease the risk of colorectal cancer in women, potentially through FAD-dependent methylenetetrahydrofolate reductase (MTHFR) [47]. Analysis of riboflavin and the risk of ovarian cancer failed to identify any association [24]. In non-smoking women, increased riboflavin intake was associated with a decrease in the risk of lung cancer [48].

While results are mixed, the role of FAD as a regulator of redox, TCA and ROS in the cell appears to influence cancer risk, which is supported by some findings indicating negative correlations between riboflavin and cancer. The present lack of clarity and mechanisms through which riboflavin may alter cancer risk suggest that additional studies on riboflavin are required to determine potential protective effects in additional cancer types and contexts. Further studies focused on the role of MAIT cells as potential mediators of antitumor responses represent another important area of exploration.

## 5. Niacin B3

Niacin (nicotinic acid) is a precursor of NAD and NADP, a cofactor utilized by multiple enzymes throughout the body. Unlike other B vitamin-derived cofactors, human cells can synthesize NAD through a number of salvage pathways that are independent of niacin. NAD is involved in numerous redox reactions where NAD (oxidizing agent) and NADH (reducing agent) are interconverted. A second role of NAD is to act as a substrate for sirtuins (see below), which mediate deacetylation reactions and also facilitate the activity of a number of ADP-ribosylation enzymes. NAD is also involved in the maintenance of genomic stability and regulation of enzymes involved in epigenetic regulation [49]. NAD-dependent enzymes are involved in base excision repair [50]. High NAD levels (NAD/NADH) inhibit ROS production [51].

The conversion of NAD to NADP by NAD kinase yields a form of the cofactor for enzymes predominantly involved in anabolic biosynthetic reactions such as fatty acid synthesis, whereas NAD is more characteristic of catabolic reactions generating energy from the TCA cycle. Niacin deficiency can result in Pellagra, a rare disease in developed countries resulting in inflamed skin, diarrhea and/or dementia. Tryptophan metabolism is a source of NAD. Diets deficient in tryptophan can also lead to pellagra or an inability to utilize niacin. Carcinoid syndrome involves gastrointestinal neuroendocrine tumors that use tryptophan as an energy source, producing large quantities of serotonin that limit availability of both niacin and tryptophan. 

Obesity, long considered a condition of metabolic imbalance, is now viewed as an inflammatory disease. A key component of obesity is the LPS activation of infiltrating macrophages in adipose tissue. In high-fat diet-fed mice, niacin increased adiponectin (hormone regulating fatty acid catabolism) gene and protein expression and decreased MCP-1, IL-1β and the frequency of activated M1 macrophage [52]. Niacin is touted for its drug-like efficacy in increasing low-density lipoprotein (LDL) and reducing serum triglyceride levels. These effects include anti-inflammatory action as shown in humans with non-ST elevated acute coronary syndrome, where hs-CRP is significantly reduced following niacin treatment [53]. In ApoE^−/−^ mice, which manifests elevated cholesterol levels, niacin inhibited the progression of atherosclerosis while suppressing inflammatory cytokines and adhesion molecules, NF-κB and apoptosis of vascular smooth muscle [54]. In a high fat diet (HFD) model of liver steatosis, niacin decreased liver fat content and oxidative products in rats. These effects were also noted in mice with pre-existing steatosis prior to niacin treatment. Niacin treatment resulted in reduced diacylglycerol acyltransferase, an enzyme in triglyceride synthesis [55]. These results have been recapitulated in humans [56]. In another study, niacin was shown to reduce liver cholesteryl ester transfer protein (CETP) expression via its effects on reducing hepatic macrophage [57]. Niacin inhibited carrageenan-activated neutrophil migration in mice further suggesting its anti-inflammatory role through mechanisms involving alterations in chemoattraction [58]. Niacin signals through GPR109A, the same receptor as the short chain fatty acid (SCFA), butyrate. Monocytes and macrophages express GPR109A. When treated with LPS (a TLR4 agonist), niacin reduced inflammatory cytokine secretion of TNF-α, IL-6 and MCP-1; these results were also observed following monocyte stimulation with heat-killed *L. monocytogenes,* TLR2 agonists. Niacin reduced the amount of NF-κB nuclear localization, chemotaxis and adhesion to human umbilical vein endothelial cell (HUVEC) and vascular cell adhesion molecule (VCAM) via integrin very late antigen 4 [59]. In rats treated with LPS to induce endotoxemia, high-dose niacin inhibited NF-κB activation and inflammatory cytokine gene expression in lungs and tissue damage, thereby increasing survival. Niacin treatment increased glutathione levels and decreased malondialdehyde levels (a marker of oxidative stress) [60]. In nephretomized rats used to model chronic kidney disease, niacin administration reduced MCP-1, plasminogen activator inhibitor (PAI-1), TGF-β, p47(phox), p22(phox), COX-1 and NF-κB activation, concomitant with reduced hypertension, proteinuria, glomerulosclerosis and tubulointerstitial injury [61].

Mice fed a diet enriched in saturated fatty acids to induce blood-brain barrier (BBB) defects and neuroinflammation, were completely protected by treatment with either niacin or nicotinamide [62]. Parkinson’s Disease patients provided low-dose niacin showed an increased M2 macrophage at the expense of the M1 macrophage, which was associated with an improved quality of life [63]. Immune activation by treatment of mice with LPS, TNF-α or IL-1 led to an increase in GPR109A expression in adipose tissue and LPS increased receptor expression in the spleen, as did lipoteichoic acid, poly dI:dC and macrophage-produced TNF-α and IL-1 [64]. GPR109A influences physiology in a tissue-dependent manner; in colonocytes, its activation is anti-inflammatory and induces apoptosis, whereas in keratinocytes niacin induces flushing via COX-2 inflammatory pathways [65]. GPR109A is expressed in normal mammary tissue but is shut off in human primary breast tumors. Furthermore, GPR109A deletion triggers earlier onset of tumors and lung metastases in an mouse mammary tumor virus (MMTV)-Neu mouse model of breast cancer [65].

Nicotinamide is a B3 vitamer that also has therapeutic activity. NAD is a substrate of the enzyme sirtuin 1 (SIRT1) involved in deacetylation reactions and poly ADP-ribose polymerase (PARP1), involved in poly ADP-ribosylation [66]. Many non-redox enzymes convert NAD to nicotinamide, including SIRT1 and PARP1 [67]. SIRT1 is an NAD-dependent enzyme that deacetylates histones and transcription factors thereby regulating gene expression, impacting DNA repair, apoptosis, cell proliferation, hormone responses and metabolism [68]. PARP1 is activated by DNA damage and DNA breaks [69]. Extreme DNA damage over-activates PARP1 thereby depleting NAD and ATP levels resulting in cell necrosis [69,70]. Interestingly, nicotinamide and NADH represses the activities of SIRT1 and PARP1 [67]. Therefore, the activity of these enzymes represent an important “sensing” mechanism of NAD/NADH ratios in the cell. 

Nicotinamide is anti-inflammatory and downregulates NF-κB via SIRT1 deacetylation [67]. A number of in vitro, ex vivo and animal studies have examined the effect of nicotinamide showing the efficacy of nicotinamide in reducing immunosuppression following UV irradiation, enhanced DNA repair in keratinocytes and melanocytes, suppression of inflammatory cytokines in keratinocytes and suppression of tumor formation in animals, reviewed in [70]. In human clinical trials, oral nicotinamide reduced the incidence of actinic keratosis, squamous cell carcinoma and basal cell carcinoma. Additional animal studies show that nicotinamide suppressed chemically induced lung tumors, liver tumors, non-lymphocytic leukemia and kidney tumors [70]. Finally, clinical trials involving Accelerated Radiotherapy with CarbOgen and Nicotinamide (ARCON) showed efficacy in treating head and neck cancers, larynx and bladder cancers, reduction in tumor hypoxia in primary colon tumors and metastasis to the liver in animal models. Nicotinamide did not show significant effects in clinical trials attempting to treat glioblastoma or non-small cell lung carcinoma [70]. However, in a 16-year follow up study, consisting of nearly 650 hepatocellular carcinoma (HCC) cases, higher niacin intake was associated with a lower risk of HCC, which displayed an inverse dose response association [71].

While evidence for a beneficial role of niacin in certain cancer types appear solid, interpretation of the mechanisms through which niacin acts is complicated as they may be attributed to NAD/P as redox cofactors, or the products generated via sirtuin degradation. It is noteworthy that an active field of research, beyond the scope of this review, involves efforts to develop inhibitors of nicotinamide phosphoribosyl transferase (NAMPT) which mediates the rate-limiting step of an NAD salvage pathway (reviewed in [72]). 

## 6. Pantothenic Acid B5

Pantothenic acid is an essential vitamin as it is required for the synthesis of CoA, a key cofactor in the TCA cycle and fatty acid metabolism. CoA is also required for acylation reactions. Deficiency in B5 is very rare as it is available in a variety of plants and animal products and therefore has not been studied in great detail. Uptake of pantothenic acid occurs via the sodium-dependent multivitamin transporter (SMVT), which also transports biotin (B7). Intestinal deletion of SMVT results in stunted growth, spontaneous and severe inflammation, increased gut permeability and early death. Surprisingly, supplementation of these mice with pantothenic acid and biotin curtailed these phenotypes [73]. These results suggest an alternative mechanism for these B vitamins’ absorption other than through SMVT. In a follow-up study, confirmation that biotin levels in SMVT-deficient mice were decreased but not eliminated supports this conjecture [74]. Given that the transporter facilitates the transport of both B5 and B7, the attribution of phenotypes to biotin vs. pantothenic acid remains obscured but suggests that one or both of these vitamins plays a role in maintaining gut homeostasis.

Greater insights as to the role of pantothenic acid have come from the study of a family of proteins encoded by vanin genes. CoA catabolism generates pantetheine that is acted upon by vanins, otherwise known as pantetheinases, which generate pantothenate and cysteamine, the latter of which potentiates inflammation [75]. Mice deficient in vanin-1 are resistant to apoptotic oxidative tissue injury caused by γ-irradiation or paraquot [76]. These mice also attenuated chemically induced inflammation in models of colitis [77]. PPARγ inhibitors blocked the anti-inflammatory phenotypes of vanin-1-deficient mice, suggesting that the activity of vanin-1 antagonizes PPARγ [77]. Exposure of human mononuclear cells to oxidative stress inducers led to an increase in vanin-1 expression and a decrease in PPARγ [78]. Studies of vanin-1-deficient mice show a reduced ability to control *Shistosoma manosoni* and *Coxiella burnetii* infections suggesting that the role of vanin-1 induces inflammatory responses to infections [77,79]. Vanin-1-deficient mice display elevated levels of reduced glutathione in multiple tissues. Cysteamine breaks disulfide bonds that inactivate proteins and directly inhibits γ-glutamylcysteine synthase, the rate-limiting step in glutathione synthesis [76]. These findings point to cysteamine as the source of oxidative stress (ROS) generated during inflammation in vanin-1-deficient mice rather than pantothenate. Cysteamine administration to vanin-1 mice restores the inflammation observed to levels comparable to wild-type mice [76,80]. 

Compared to other B vitamins, the direct effects of vitamin B5 supplementation are scant. Therefore, the impact of B5 must be gathered through its known metabolism that has thus far highlighted cysteamine’s pro-inflammatory properties. Given the importance of CoA, additional studies appear warranted to determine their effect on inflammatory processes and cancer. Analysis of pantothenate in the absence of pantetheinase activities, while challenging, may shed light on the more subtle roles of pantetheine in human health. 

## 7. Pyridoxine B6 

Despite the simplicity of pyridoxine synthesis (Figure 1), conversion of pyridoxin to its associated vitamers in human cells is complex. Pyridoxin (PN) and its vitamers pyridoxal (PL) and pyridoxamine are phosphorylated by pyridoxal kinase (PDXK) to generate pyridoxin-5′-phosphate (PNP), pyridoxol-5′-phosphate (PLP) and pyridoxamine-5′-phosphate (PMP). Each form of B6 are present in various foods; however, before absorption, they must be dephosphorylated by intestinal alkaline phosphatase [81]. Among these forms of vitamin B6, PN and PLP have received the greatest amount of attention, due to their more significant impact on human processes.

Vitamin B6-derived metabolites (PLP and PMP) serve as interconverting cofactors (transaminase) for >140 enzymatic reactions and have been estimated to be essential for 4% of all enzyme activities in the human genome. These reactions are important for amino acid, carbohydrate and fatty acid metabolism, and neurotransmitter production. PLP is a cofactor of decarboxylases and racemases, where PLP is not converted to PMP. PLP acts as a cosubstrate (phosphate donor) for glycogen phosphorylase.

Several studies have demonstrated the bioactivity of B6 and its vitamers in cancer models in vitro as inhibitors of cell proliferation, enhancement of chemotherapy cytotoxicity and alterations in gene expression. PN inhibits the growth of both murine and human melanoma cell lines and in this context PL displayed substantially greater effects than PN [82]. These observations have been extended to additional cell lines including hepatoma HepG2 and gastric cancer MKN45 cells [83], MCF-7 breast cancer and PANC-1 human pancreatic cancer cells [84]. The inhibition of cell proliferation in these models was assumed or verified as being correlated with elevated PLP.

Studies of PN and PLP in animal models of cancer have yielded mixed results. Some studies have reported an inverse correlation between PLP and different cancers [85]. In an examination of bile acids that are a risk factor for developing colorectal cancer (CRC), B6 supplementation increased fecal mucin levels and reduced the ratio of lithocholic acid to deoxycholic acid in a dose-dependent manner [86]. A study of rats exposed to 1,2-dimethylhydrazine to induce intestinal damage observed that vitamin B6 supplementation reduced the expression of alkaline phosphatase, a marker of intestinal damage and reduced cell proliferation [87]. Vitamin B6 was shown to stimulate proliferation of blood and splenic lymphocytes [88]. Using BALB/c mice fed varying doses of B6, very high doses of B6 (7.7 and 74.3 mg/day) exhibited the greatest impact on tumor volume reduction. Strong negative correlations were noted for PLP and tumor volume in these mice that also exhibited increased lymphocyte proliferation [88]. These results do not allow clear discrimination between whether the tumor inhibition following B6 feeding is due to an intrinsic effect on tumor growth or, alternatively, a greater stimulation of the antitumor immune response. 

Treatment of A549 human non-small lung carcinoma (NSCLC) cells with a combination of PN and cisplatin potentiated the cytotoxicity of cisplatin by increasing the intracellular accumulation of cisplatin [85,89]. These studies concluded that PDXK activity was important for the observed enhancement of cisplatin. This is consistent with a report indicating that PDXK expression was positively correlated with overall survival of NSCLC patients treated with cisplatin. Treatment of human colon cancer and HEPG2 cells with 0.5-mM PL increased the expression of the cyclin inhibitor p21 as the result of p53 repression [90]. Similarly, PL treatment of HT29 colon cancer and HEPG2 cells increased the expression of insulin growth factor binding protein 1, a tumor suppressor [84]; the same finding was reported in MCF-7 breast cancer cells [84]. 

Reduced dietary intake of vitamin B6 has been documented as a risk factor for developing cancer [85,91]. Other studies have noted an inverse correlation between some cancers and vitamin B6 and/or serum PLP levels [85,92]. These include pancreatic, gastric adenocarcinoma, prostate, oral/pharyngeal, lung and colon cancers. It has been shown that B6 deficiency decreases the activity of serine hydroxymethyltransferase (SHMT) and betaine-homocysteine methyltransferase (BHMT), which reduce the pool of methylene groups for 5,10-methylene-THF resulting in an increase in the frequency of uracil incorporation during DNA synthesis that may be associated with mutation, and the DNA strand breaks [93]. The extent that this promotes carcinogenesis remains only partially characterized. In patients with non-small cell lung carcinoma, elevated levels of PDXK was identified as a good prognostic marker [92]. A meta-analysis of studies examining vitamin B6 in renal cell carcinoma patients concluded that B6 was protective and a useful biomarker; however, variability across studies made these conclusions tentative [45]. In a similar analysis of breast cancer risk involving nearly 50,000 cases, vitamin B6 levels were borderline significant in estrogen/progesterone receptor-negative tumors but not ER/PR + tumors [44]. Finally, an assessment of esophageal cancer found that an increase of 1 mg/day of vitamin B6 corresponded to a 16% reduction in cancer risk [94]. Hand–foot syndrome (HFS) is a dermatological reaction to toxicities generated by some cancer chemotherapies. Pyridoxine has been used to prevent HFS, although a meta-analysis of studies examining the efficacy of pyridoxine to prevent HFS found no significant differences in prevention of low-grade HFS compared to placebo controls; however, higher doses of B6 (400 mg daily) were statistically preventative [95]. 

## 8. Biotin B7

While biotin is available in many foods that are part of a standard diet, it is usually presented as a protein-bound form of biotin that is not readily available to cell metabolism. Peptide- or lysine-bound (biotinyl-lysine) biotin is released by the action of the pancreatic enzyme biotinidase and transported into cells by a number of different transporters [96]. Once inside the cell, biotin is covalently bound to biotin carboxyl carrier protein (BCCP) which serves as a prosthetic group for a number of carboxylases/decarboxylases regulating gluconeogenesis, lipogenesis, fatty acid synthesis and catabolism of branched-chain amino acids as well as valine and isovalerate. Biotinyl-AMP represents the activated form of biotin that is formed in the course of BCCP biotinylation. Biotin plays an indirect role in regulating gene expression as it has been reported that deprivation alters gene expression of multiple genes in the liver [97], including NO-like functions that increase cGMP via increased guanylate cyclase [98]. Biotin deficiency stems primarily from genetic changes in biotinidase which is required for cleavage from BCCP and recycling, resulting in alopecia, delays in development, seizures, aciduria and others neurological conditions. Biotin deficiency is also associated with elevated inflammation [99].

Neurodegenerative diseases are associated with mitochondrial dysfunction and oxidative stress and cell death. Myelin producing oligodendrocytes treated with biotin partially restored mitochondrial function, reduced oxygen free radicals and apoptosis [100]. Monocyte-derived DCs cultured in biotin-deficient medium produced elevated inflammatory cytokines in response to an LPS challenge [99]. In a study of monozygotic twins discordant for body mass index (BMI), BMI was anticorrelated with serum biotin levels and inflammation and hypertriglyceridemia [101]. 

Biotin deficiency impacts the expression of transcription factors such as NF-κB and SP1/3 highlighting the role of biotin in regulation of immunological phenomenon in a manner outside of its carboxylation/decarboxylation function [102]. While the molecular targets of biotin have been studied in some detail, there is much still to learn about the role of biotin in immune regulation and cancer and at present remain highly understudied.

## 9. Folate B9 

Folate is transported into human cells as a monoglutamate form of the vitamin. Once inside the cell, tetrahydrofolate polyglutamates are formed, representing the active form of the vitamin. Concerns over meeting the daily minimum requirements for folate has led to the fortification of a variety of foods—e.g., cereals. This practice has led to an appreciable decrease in developmental problems associated with neural tube defects where folate plays an important role [103]. While the literature clearly illustrates the detrimental effects of folate deficiency, the metabolism of rapidly dividing malignant cells generate additional requirements for folate, thereby drawing into question whether folate supplementation may do more harm than good in this context. 

There is a general lack of literature examining the role of folate in the regulation of the immune system; however, its role as a key regulator of one-carbon metabolism is well-studied and described in a subsequent section. Both folate and vitamin B12 deficiency are associated with the reduction in glutathione, a major antioxidant protein in the cell. Azoxymethane (AOM) is used in many models to induce tumors and to induce oxidative stress. AOM-treated rats display oxidative stress, glutathione depletion, oxidation of proteins and oxidative damage of DNA. Folate and B12 reduced oxidative stress and the cytotoxic effects of AOM in mice [104]. Folate deficiency has been shown to have negative consequences for some immune functions. Treatment of CD8^+^ T cells and phytohaemaggutinin and IL-2 promotes cell proliferation but stimulation was inhibited when folic acid was absent. This activity did not affect CD4^+^ T cells [105]. Folate deficiency in primary human lymphocytes reduced cell proliferation and resulted in increased DNA strand breaks, apoptosis and cell cycle arrest [106]. Folate deficiency has also been linked to reduced DC maturation and effector functions including: reduced IL-2, TNF-α, IL-6 and IL1-β in response to LPS stimulation. These changes were coupled to reduced CD4^+^ T cell differentiation with reduced Th1 and Treg cell populations [107]. Anti-inflammatory Treg cells express the folate receptor constitutively and at high levels. Blockade of this receptor leads to reductions in Treg cell populations [108]. Finally, high oral doses of folic acid reduced the inflammatory response of mice with allergic dermatitis through inhibition of T cell cytokine secretion [109]. 

In mice implanted with tumor cells derived from various MMTV-Wnt breast tumors, differential effects of folic acid withdrawal were observed in a comparison of non-metastatic epithelial, non-metastatic mesenchymal, or metastatic mesenchymal cell lines. After 72-h growth in the absence of folic acid, the non-metastatic mesenchymal cell line displayed the largest change in its transcriptome. The type I interferon signaling pathway was activated under folic acid insufficiency. This pathway is downregulated in aggressive triple-negative breast cancers [110]. This differential response is consistent with other data indicating that folic acid supplementation impacts cell migration and metastasis. In a similar study using nasopharyngeal epidermoid carcinoma cell growth in folate-deficient medium, hypermethylation of H-cadherin promoter sequences were observed, which corresponded to reduced expression [111]. 

A number of in vitro and animal studies have shown that folate deficiency may drive oncogenic events, whereas high folate intake promotes the growth and progression of established tumors [112]. Low folate intake has been associated with increased expression of immune-related genes, urokinase and iNOS, and the downregulation of genes encoding adhesion proteins—protocadherin-4, nidogen and integrin αV. Gene expression changes in the intestine were pronounced in aged rats fed a folate-deficient diet including reduced expression of urokinase, p53, insulin-like growth factor binding protein-3 and the vav-1 oncogene [113]. Using three normal colonic cell lines with varying doses of folate, gene expression was altered including the upregulation of folate receptor-1 which was inversely proportional with folate levels. Oncogenes p53, p21, p16 and β-catenin followed a similar pattern. DNA strand breaks were increased with decreased folate. Other changes included the Wnt/APC pathway and genes involved in cell adhesion, migration and invasion [114]. Folate antagonists, methotrexate and trimetrexate treatment of glioma cell lines reduced cell migration but had no impact on invasion potential [115].

A study of a small number of women at high risk for developing breast cancer (BRCA1/2) found that high plasma levels of folate were associated with high risk [116]. Other studies come to an opposing conclusion indicating a protective effect of folic acid supplementation in women with BRCA1 mutation [117]. No such association was attributed to B6 or B12 [116]. Epidemiological and clinical studies indicate that folate intake and blood levels are both inversely correlated with colorectal cancer risk. The risk reduction for CRC is substantial when comparing the upper and lower quartiles of folate intake, corresponding to a 40% reduction [118]. High serum folate in men with prostate cancer was associated with elevated cell proliferation [119]. Similarly, folate intake was positively associated with risk of recurrence in patients with non-muscle-invasive bladder cancer [120]. Despite these findings, reports of serum folate in hepatocellular carcinoma patients were found to be inversely associated with tumor size, tumor multiplicity and metastasis [121]. Interestingly, serum folate levels were reduced with HCC stage progression. Using an HCC xenograft mouse model, folate-deficient feeding promoted tumorigenesis and metastasis [122]. Similarly, folate deprivation of cultured epithelial colon cancer cells enhanced cell migration and invasion resembling an epithelial–mesencymal transition, associated with snail gene expression and E-cadherin repression with increased expression of β1 integrin and proteolysis activity of MMP2. These patterns were abolished by the inhibition of sonic hedgehog due to the hypomethylation of its promoter or NFκB [123]. High folate intake is inversely associated with ovarian cancer risk [124]. Carcinomas have been reported to over-express folate receptor α. Ovarian tumors over-express folate receptor α but display a decreased expression of another transporter, reduced folate carrier. These outcomes were associated with folate receptor α gene amplification and hypermethylation of the reduced folate carrier. Folate promoted proliferation, migration and invasion in vitro. Knock down of folate receptor α or over-expression of the reduced folate carrier negated these effects. In summary, folate deficiency is negatively correlated with cancer risk for a number of malignancies; however, there is increasing concern that folate supplementation in individuals with an already developed cancer may further drive cancer progression. The major role of folate relates to one-carbon metabolism which is discussed below.

## 10. One-Carbon Metabolism

**Figure 3:**
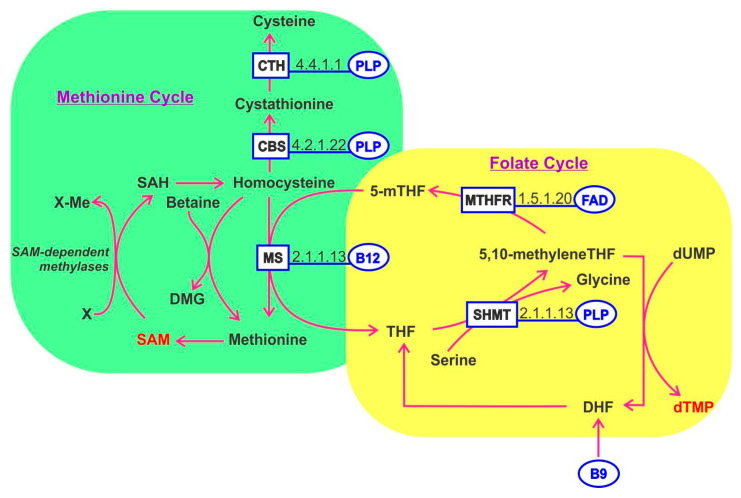
The methionine and folate cycle pathways involving PLP-, FAD- and B12-dependent enzymes. The folate cycle begins with the conversion of dietary folate (B9) into dihydrofolate (DHF), which is then reduced to tetrahydrofolate (THF) by the enzyme dihydrofolate reductase (DHFR). THF is next converted to 5,10-methyleneTHF by serine hydroxymethyltransferase (SHMT), a reaction that is coupled with the hydroxylation of serine (Ser) to glycine (Gly) and requires PLP as a cofactor. Thymidylate synthase (TS) uses 5,10-methyleneTHF as a methyl donor to methylate deoxyuridine monophosphate (dUMP), creating deoxythymidine monophosphate (dTMP). This step regenerates DHF for continued cycling. Alternatively, 5,10-methyleneTHF can be reduced by methylenetetrahydrofolate reductase (MTHFR) to 5-methytetrahydrofolate (5-mTHF) using FAD as a cofactor. As part of the methionine cycle, 5-mTHF donates a methyl group to regenerate methionine from homocysteine (Hcy), which is catalyzed by methionine synthase (MS) that requires B12, in the form of methylcobalamin, as a cofactor. To generate the methyl donor *S*-adenosylmethionine (SAM) for use by multiple methyltransferases. SAM is demethylated during the methyltransferase reactions to form *S*-adenosylhomocysteine (SAH) which is then hydrolysed by *S*-adenosylhomocysteine hydrolase (AHCY) to form Hcy. Hcy can also enter the trans-sulfuration pathway catalyzed by cystathionine beta synthase (CBS) and cystathionine gamma lyase (CTH), both requiring PLP as a cofactor, to create cysteine.

One-carbon metabolism refers to a complex network of biochemical pathways involving homocysteine, methionine and B vitamins (B2, B6, B9 and B12). The folate cycle and methionine cycle are coordinated and mediate diverse cellular processes including: DNA synthesis (purines and thymidine), energy (ATP), redox potential (NADPH) and lipid, polyamine, amino acids and phospholipid biosynthesis (Figure 3). Importantly one-carbon metabolism generates *S*-adenosylmethionine (SAM) which is a universal methyl donor for methylation of DNA and RNA and histones, thereby regulating transcription and translation. 

Inputs for the folate cycle include serine and glycine. The folate cycle is segregated in the cytoplasm and the reactions occurring in the mitochondria. The methionine cycle requires methionine to generate cysteine, a key component of a third pathway referred to as trans-sulfuration, which generates glutathione—a mediator of redox balance, inhibitor of ROS-induced oxidative stress and which generates SAM. An exciting research area considers that maternal intake of B vitamins involved in one-carbon metabolism may impact the long-term cancer risks of offspring through modulation of epigenetic imprinting. While several studies have reported evidence in this direction, the field is fraught with contradictory findings at this stage and is therefore considered too premature for detailed description here [125]. Folate and cobalamin deficiency can alter the balance of one-carbon metabolism pathways. PLP, derived from vitamin B6, plays an essential role as a cofactor for enzymes in homocysteine metabolism, encompassing both folate-mediated one-carbon metabolism and the trans-sulfuration pathway. As such, vitamin B6 is involved in purine, thymidylate and methionine synthesis. These activities have highlighted vitamin B6′s potential role in genome stability and epigenetic regulation [126]. Glutamylated folate is involved in the carriage and transfer of one-carbon units (formate) in the form of 10-formyltetrahydrofolate, required for purine biosynthesis, (formaldehyde) in the form of methylenetetrahydrofolate required for thymidylate biosynthesis or (methanol) in the form of 5-methyltetrahydrofolate, required for methionine and SAM biosynthesis. Each of these forms serves as a cofactor for one or more enzymes. Folate functions as a coenzyme in numerous reactions involving one-carbon transfer in nucleotide biosynthesis, amino acid metabolism. The action of folate and B12 are linked since methylcobalamin is needed to regenerate tetrahydrofolate from methyltetrahydrofolate. Tetrahydrofolate is a key factor serving as a methyl donor in multiple pathways. B12 deficiency leads to the trapping and accumulation of methyltetrahydrofolate disrupting the normal cycling of cofactors [127].

Many tumors become methionine-dependent, unable to proliferate in the absence of exogenous methionine even when its precursor homocysteine is available. The conversion of homocysteine to methionine is carried out by methionine synthase which transfers a methyl group from 5-methyl THF in the presence of cobalamin. Detailed understanding of methionine dependence of tumors is an area of active research to identify potential novel drug targets. Tumor spheres of glioblastoma are methionine-dependent due, in part, to disruptions in folate and one-carbon metabolism. One-carbon metabolism balances the flux of one-carbon units toward either the folate cycle that generates nucleotides for DNA and RNA synthesis and energy metabolism or alternatively, toward the methionine cycle for methionine generation where it serves as precursor of SAM, which is required for the methylation of DNA, RNA, histones and proteins impacting gene expression (reviewed in [128]). Vitamin B12 deprivation results in a decrease in SAM concentrations and strong reductions in cancer cell growth [129]. Hyperhomocysteinemia is a risk factor for cardiovascular disease. Using apoE-null mice provided a diet rich in methionine but depleted in vitamins B6, B9 and B12, increased cardiovascular signatures were found to develop in said mice, including elevated expression of the receptor for advanced glycation products, VCAM-1 and MMP-9 in the vasculature; provision of the B vitamins alleviated these effects [130]. By contrast, a human clinical trial where patients who experienced ischemic attack or stroke were provided vitamins B6, B9 and B12 to lower homocysteine levels; while such treatments indeed lowered homocysteine levels, markers of vascular inflammation were unaffected [131]. 

A meta-analysis of one-carbon metabolism-related vitamins (B6, B9 and B12) concluded that there was no association between B12 and breast cancer risk [44]. In another meta-analysis study examining methylenetetrahydrofolate reductase polymorphisms (MTHFR C677T) and B9 and B12 intake concluded that low intake of folate was associated with increased risk of breast cancer, whereas no association were found for B12 [132]. A study seeking to find genetic associations between vitamins and five cancers also concluded that B12 was not relevant to colorectal, breast, prostate, malignant melanoma or squamous cell carcinoma [133]. A study of B vitamins involved in one-carbon metabolism in the setting of esophageal cancer found that vitamin B2 and B12 were positively correlated with cancer risk, whereas vitamin B6 and B9 were inversely correlated [94]. From an analysis of nearly 4000 incident lung cancer cases, B6 intake was deemed protective, whereas B9 and B12 intake were not associated with lung cancer risk [134]. In a large meta-analysis of patients (757,185 subjects) for all cancers, elevated B12 was identified as a risk factor for cancer; this was particularly evident in liver, pancreatic and myeloid malignancies [135]. Finally, a meta-analysis of prostate cancer risk found that both vitamin B9 and B12 were positively associated [136].

## 11. Cobalamin B12

B12 absorption requires release of the vitamin from dietary proteins. Once released it may bind to gastric intrinsic factor which is absorbed in the gastrointestinal tract (ileum) through a receptor-mediated process. Individuals that do not produce adequate quantities of intrinsic factor suffer from pernicious anemia due to the low bioavailability of cobalamin. Additionally, certain medicines can interfere with B12 absorption including chloramphenicol, metformin, proton pump inhibitors and histamine. B12 absorption is saturable and relatively inefficient as ~1% of dietary B12 is absorbed and the remainder is excreted in urine. 

Vitamin B12 is important in the regulation of select immune cell types, including NK cells and CD8^+^ T cells through an upregulation of their expansion [137]. In aged rats, B12 deficiency was associated with a decline in NK cell activity and a reduction in a subset of B lymphocytes [138]. Interestingly, this study also highlighted the importance of maintaining normal levels of folate and B12, since the age-induced B12 reductions were most evident when folate was supplemented. NK cell activity was compromised in association with free unmetabolized folic acid illustrating the importance of achieving a proper balance between folate and B12 levels. 

High serum cobalamin levels were associated with poor survival in patients with HCC [139]. A study of non-small cell lung carcinoma examined plasma levels of cobalamin and cobalamin carrier proteins—transcobalamin, holotranscobalamin and haptocorrin. Among the analytes examined, significant associations were determined only for haptocorrin that was significantly elevated in patients with NSCLC, suggesting that this may be a useful biomarker for diagnostic purposes [140]. Similarly, in a case-control study of prostate cancer, B12 and holo-haptocorrin levels were positively associated with cancer risk [141]. In a nested case-control study, blood B12 levels were positively associated with overall lung cancer risk [142].

## 12. Conclusions

The accumulated knowledge of B vitamins and their vitamers as cofactors to a large number of essential enzymes and cellular activities is impressive. Overall, evidence for some B vitamins having a protective role in various cancers remains elusive, begging the question, why? The answers are likely to be complex, owing to the number of variables associated with any B vitamin such as absorption, bioactivity and variability of vitamer generation to name a few. B vitamins influence the function of such a wide spectrum of cell functions, so it should perhaps not be surprising that studies aimed at assessing their role as regulators of immune function and cancer are variable. It may not be adequate to simply interogate B vitamin intake or even blood concentrations of B vitamins. Animal studies allow a more direct assessment of individual B vitamers and in some cases give greater clarity of the beneficial and detrimental effects of B vitamin consumption. Ultimately, these results are very challenging to translate to humans in the form of intervention studies since the design of cancer prevention studies would require compliance for many years in very large cohorts. Alternatively, human studies in the future may require more comprehensive molecular analyses of B vitamins and their corresponding vitamers to be coupled with a high-resolution analysis of a variety of immune functions and tumor subtypes that may allow stronger and more convincing associations to be uncovered. As appears to be the case for folate (B9), B vitamins may provide protection from developing cancer but play a detrimental role in patients diagnosed with cancer. This single example suggests that a more detailed and sophisticated analysis of B vitamins will be of high potential importance.
